# Does the fairness of healthcare resource allocation affect utilization efficiency?—an empirical study based on China's provincial panel data

**DOI:** 10.3389/frhs.2025.1409421

**Published:** 2025-05-27

**Authors:** Anying Xu, Haibin Wei

**Affiliations:** ^1^ School of Artificial Intelligence and Information Technology, Nanjing University of Chinese Medicine, Nanjing, Jiangsu Province, China; ^2^ Department of Development Planning (Office of Discipline Development, ‌Department of Hospital Management and Social Services), Guangxi University of Chinese Medicine, Nanning, Guangxi Zhuang Autonomous Region, China

**Keywords:** healthcare resources, Theil index, data envelopment analysis, Tobit model, utilization efficiency

## Abstract

**Objective:**

This study aims to measure the fairness and efficiency of healthcare resource allocation in 31 provinces and cities in China from 2014–2019 and explore the relationship between the fairness and efficiency of healthcare resource allocation in China.

**Methods:**

The Theil index and data envelopment analysis are used to evaluate the fairness and efficiency of healthcare resource allocation in the studied provinces and cities from 2014–2019. The DEA method, including the BCC model for static efficiency analysis and the Malmquist index for dynamic efficiency analysis, provides a comprehensive assessment of healthcare resource allocation efficiency. The Tobit model is employed to analyze the influencing factors.

**Results:**

The measurement results of fairness indicate that regional disparities are the main factor contributing to the unfairness of healthcare resource allocation in China. Among them, the regional differences in total assets are more pronounced. The results also show that the overall efficiency of healthcare resource allocation has improved during the period of 2014–2019, and low technological level is a major factor affecting the efficiency. Furthermore, considering the dimensions of regional development level, total healthcare resource quantity, and fairness in resource allocation, the study reveals that population density, urbanization rate, number of beds, total assets, fairness in the allocation of healthcare technical personnel, and fairness in the allocation of total assets significantly impact the efficiency of healthcare resource allocation in China.

**Conclusion:**

The fairness of healthcare resource allocation does have certain impacts on the utilization efficiency of healthcare services. The most important finding is that the fairness in the allocation of healthcare technical personnel has the highest positive impact on the efficiency of healthcare resource allocation. Additionally, the fairness in the allocation of total healthcare assets may decrease its utilization efficiency to some extent. Based on these findings, corresponding improvement strategies and recommendations for optimizing healthcare resource allocation are proposed.

## Introduction

Fairness and efficiency embody the values of healthcare system reform, requiring long-term policy attention and guidance from the government. Healthcare resources refer to the general term for various production factors that are occupied or consumed in the process of providing healthcare services, while healthcare resource allocation refers to the study of how to allocate and use healthcare resources reasonably and effectively (1). Healthcare resources are closely related to people's rights and interests in life and health. The issue of “difficulties in seeing a doctor and expensive medical treatment” is closely related to the current unreasonable allocation of healthcare resources in the form of an “inverted triangle”. Under a series of policy interventions, the problems of “difficulties in seeing a doctor and expensive medical treatment” have been somewhat alleviated. However, due to the scarcity and agglomeration effects of healthcare resources, as well as information asymmetry and adverse selection issues, the healthcare service market is prone to imbalanced allocation of resources (2), manifesting as a dual issue of both unfairness and inefficiency (3). With the increasing contradiction between the demand for high-quality healthcare resources by the people and the imbalanced allocation and insufficient supply of healthcare resources becoming more prominent (4), the imbalance in the allocation of healthcare resources and the inefficient operation of the medical system have become key livelihood issues that the Chinese government needs to address (5). Therefore, how to balance fairness and efficiency in the process of healthcare resource allocation remains a key issue that requires the government's attention. Fairness and efficiency highlight the institutional logic of government healthcare service supply, which can effectively measure the effect of healthcare resource allocation. Efficiency reflects the production and utilization status of social wealth, indicating the degree to which resources meet human needs under given inputs and technological conditions (6). Fairness reflects the distribution of social wealth and serves as a measure of the rationality of interests among individuals (7); whether social wealth is fairly distributed will to some extent affect its production and utilization. Therefore, fairness and efficiency embody the value logic and action logic of public services (8). Healthcare resources are an important part of public resources, and healthcare services are integral units of public services. Thus, from a deeper perspective, efficiency and fairness reflect the government's perspective on justice in the production and distribution of public goods; the fairness in the allocation of healthcare resources embodies the principle of equal access to healthcare services for all citizens, while the efficiency in the utilization of healthcare services reflects the concept of procedural and outcome fairness. Consequently, fairness in healthcare resource allocation underscores the rational distribution of healthcare resources, which is crucial for ensuring fairness in access to healthcare services and reducing injustices in the allocation and utilization of healthcare resources. It represents a specific manifestation of the government's commitment to providing “basic healthcare services for all”. In terms of the relationship between fairness and efficiency, there is a strong dialectical consensus in academia. It suggests that the government should emphasize efficiency while also focusing on fairness, and when pursuing fairness, it should prioritize efficiency (9). Scholars such as Adam Smith and John Stuart Mill have argued that the fairest provision of public goods is also the most efficient (10). Aktaş et al. (11) proposed a management-oriented decision support model to help health system managers improve the efficiency of their systems. However, the current theoretical consensus is unable to accurately respond to the extent to which the fairness in the allocation of healthcare resources affects the efficiency of healthcare service utilization. Moreover, discussing the relationship between efficiency and fairness in isolation from external factors does not align with reality.

Fairness and efficiency do not operate in a vacuum with a simple one-way relationship of mutual exclusion. It is more theoretically and practically significant to delve into the multiple influencing factors. The supply of healthcare resources currently faces an overall shortage (12, 13), and in order to achieve the long-term goal of “Healthy China 2030”, the accessibility and affordability of healthcare services must be considered (14). Starting from the actual social production, the level of economic development is the main influencing factor of the total allocation of healthcare resources (15), leading to disparities in the fiscal capacity of regional governments and resulting in differences in basic public services (16). This ultimately leads to a situation where developed regions have a relative surplus of healthcare resources while underdeveloped regions face shortages in resource allocation (17). Additionally, considering that human and technological resources have specific cultivation and development cycles, the total amount of healthcare resources does not necessarily increase linearly with social and economic development (18). Population density and urbanization rates also have a profound impact on the fairness of healthcare resource allocation (19). Existing empirical research by scholars has shown that the disparities in the supply of basic healthcare resources in China decrease as the per capita GDP differences decrease. Furthermore, the smaller the population size in urban areas, the faster the disparities in basic healthcare resources decrease along with the per capita GDP differences (20). Moreover, it is worth noting that the Gini coefficient for healthcare is greater than the Gini coefficient for income distribution, indicating greater inequality in healthcare than in income distribution (21). Therefore, assessing the relationship between the fairness and efficiency of healthcare resource allocation requires a comprehensive consideration of differences in economic development, urban-rural structures, population sizes, and other factors.

With the expansion of the older adult population and the implementation of the multi-child policy, the government should pay more attention to the balance between fairness and efficiency in the allocation of healthcare resources. In addition, sporadic outbreaks of the novel coronavirus epidemic have severely affected the flow and sharing of healthcare resources, exposing the problem of uneven distribution of healthcare resources in China and highlighting the inadequacy of healthcare service capacity. Since the 18th National Congress, the Party Central Committee with Comrade Xi Jinping at its core has emphasized “ensuring the health of the people in a strategic position of prioritized development” and stated that “without the health of all the people, there can be no comprehensive well-off society (22)”. In September 2020, General Secretary Xi Jinping chaired a symposium with experts in the fields of education, culture, healthcare, and sports, where he pointed out the need to “accelerate the improvement of the quality and service level of healthcare supply, and achieve higher quality, more efficient, fairer, sustainable, and safer development of the economy and society (23)”. Therefore, based on a scientific assessment and estimation of the fairness and utilization efficiency of the allocation of healthcare resources in China, this paper intends to explain the impact of economic development level, total healthcare resources, and fairness of healthcare resource allocation on the utilization efficiency of healthcare services.

## Methods

### Fairness in healthcare resource allocation

#### Selection of indicators

To analyze the fairness of healthcare resource allocation in 31 provinces and cities in China from 2014–2019, this study selects indicators from three aspects: human resources, material resources, and financial resources. Specifically, the number of health technical personnel is selected as an indicator of human resources evaluation. In addition, the number of practicing (assistant) physicians and registered nurses is also selected as measures of healthcare human resources to analyze their internal structural differences. The number of beds is chosen as an indicator of material resources (24). The total assets are selected as an indicator of financial resources (25). The specific indicators are shown in Table 1.

**Table 1:** Indicators of healthcare resource allocation in China.

A set of indicators	Indicator	Symbol	Unit
Healthcare Human Resource	Number of Health Technical Personnel	X1	Person
	Number of Practicing (assistant) Physicians	X2	Person
	Number of Registered Nurses	X3	Person
Healthcare Material Resource	Number of Beds	X4	Bed
Healthcare Financial Resource	Total Assets	X5	Ten thousand yuan
Healthcare Output Resources	Total Healthcare Expenses	X6	100 million yuan
	Bed Utilization Rate	Y1	%
	Number of Outpatient and Emergency Visit	Y2	Person
	Number of Discharge	Y3	Person

#### Ndata source

The research data in this section comes from the “China Health Statistics Yearbook” and the “China Statistical Yearbook” from 2015–2021, obtaining panel data for 31 provinces and cities in China (excluding Hong Kong, Macao, and Taiwan) from 2014–2019. According to the classification standards of the National Bureau of Statistics, the 31 provinces and cities in China are divided into eastern, central, and western regions (26).

#### Theil index

The Theil Index, also known as “Theil's Entropy Measure”, was first proposed by Dutch economist Theil in 1967. The Theil index uses the concept of entropy from information theory to calculate income inequality and is named for this. It is an important indicator for measuring the degree of regional economic development balance (27). The Theil index can measure differences within regions and between regions. The higher the value, the more unfair the income distribution or the greater the degree of difference. The calculation formula is as follows (28, 29):

The Theil index $T_{i}$ of the disparity in healthcare resource allocation among provinces and cities in the eastern, central, and western regions is:

(1) $$T_{i}=\sum_{j}\frac{Y_{ij}}{Y_{i}}\ln\frac{Y_{ij}/Y_{i}}{W_{ij}/W_{i}}$$

The Theil index $T_{b}$ of the disparity in healthcare resource allocation among the eastern, central, and western regions is:

(2) $$T_{b}=\sum_{j}\frac{Y_{i}}{Y}\ln\frac{Y_{i}/Y}{W_{i}/W}$$

The Theil index *T* of the disparity in healthcare resource allocation on a provincial and city level is:

(3) $$T=\;T_{w}+T_{b}=\sum_{j}\frac{Y_{i}}{Y}T_{j}+T_{b}$$

Where, $T_{i}$ represents the inequality in healthcare resource allocation among provinces and cities within the eastern, central, and western regions. $T_{b}$ represents the inequality in healthcare resource allocation among the eastern, central, and western regions themselves. *T* represents the overall inequality in healthcare resource allocation at the provincial and city level. $Y_{ij}$ is the amount of healthcare resources in the *j*-th province or city within the *i*-th region, $Y_{i}$ is the total amount of healthcare resources in the *i*-th region, $W_{ij}$ is the population or weight of the *j*-th province or city within the *i*-th region, $W_{i}$ is the total population or weight in the *i*-th region, *W* is the total population or weight across all regions.

### China's healthcare resource allocation efficiency

#### Indicator selection

In order to analyze the efficiency of healthcare resource allocation in 31 provinces and cities in China, this study will select indicators from two aspects: healthcare inputs and output resources. Based on extensive literature review (30, 31), the selected input indicators include the numbers of health technical personnel, practicing (assistant) physicians, registered nurses, hospital beds, and total healthcare expenses; the output indicators include bed utilization rate as a measure of the efficiency of healthcare institutions' work, and the number of outpatient visits and discharges as measures of service efficiency. The specific indicators are shown in Table 1.

#### Data source

The research data in this section comes from the “China Health Statistics Yearbook” from 2015–2021, obtaining panel data from 31 provinces in China (excluding Hong Kong, Macao, and Taiwan) from 2014–2019.

#### BCC model

In the DEA model methodology, the classic models include the CCR model and the BCC model. The CCR model is based on the assumption of constant returns to scale, which means that when a certain number of inputs is increased, the outputs also increase in the same proportion, ultimately yielding efficiency values. On the other hand, the BCC model is based on the assumption of variable returns to scale, where increasing a certain number of inputs may result in an increasing or decreasing trend in outputs, leading to the calculation of efficiency values (32). It minimizes inputs for given outputs, suits stable and easy-to-get input data, identifies resource redundancy, gives concrete optimization advice, and is widely used in public resource allocation with its effectiveness well-proven. This study selects the input-oriented BCC model to calculate the static efficiency of healthcare resource allocation in various provinces, autonomous regions, and municipalities directly under the central government of China from 2014–2019. The model calculation formula is:

(4) $$\begin{array}{rl} & \min\left[\theta -\varepsilon \left(\sum_{i=1}^{m}s_{i}^{-}+\sum_{r=1}^{s}s_{r}^{+}\right)\right]=vd(\varepsilon )\\ & s.t.\left\{\begin{array}{c} \sum_{\;j=1}^{n}\lambda_{j}x_{j}+s_{i}^{-}=\theta x_{0}\;\\ \sum_{\;j=1}^{n}\lambda_{j}y_{j}-s_{r}^{+}=y_{0}\;\\ \sum_{\;j=1}^{n}\lambda_{j}=1\;\\ \theta ,\lambda_{j},s_{i}^{-},s_{r}^{+}\geq 0\; \end{array}\right\} \end{array}$$

In formula (4), θ represents the efficiency value of the decision-making unit, *s_i_^−^* and *s_r_^+^* are slack variables. The efficiency value result is the overall technical efficiency (TE), which can be further decomposed into scale efficiency (SE) and pure technical efficiency (PTE), where irs, -, and drs respectively represent increasing, constant, and decreasing returns to scale (33).

#### Malmquist index model

In order to further analyze the efficiency changes of decision-making units over time, this study introduces the Malmquist index model. It is often used to measure the changes in total factor productivity of decision-making units across different time periods (34). The expression for the Malmquist index (35) is:

(5) $$M(x^{t+1},y^{t+1},x^{t},y^{t})={\left[\frac{D^{t+1}(x^{t+1},y^{t+1})}{D^{t}(x^{t},y^{t})}\times \frac{D^{t}(x^{t+1},y^{t+1})}{D^{t+1}(x^{t},y^{t})}\right]}^{\frac{1}{2}}$$

The change in technical efficiency can be decomposed into pure efficiency change and scale efficiency change, the formula is as follows:

(6) $$Effch=\frac{D^{t+1}(x^{t+1},y^{t+1})}{D^{t}(x^{t},y^{t})}$$

(7) $$Tech={\left[\frac{D^{t}(x^{t+1},y^{t+1})}{D^{t+1}(x^{t+1},y^{t+1})}\times \frac{D^{t}(x^{t},y^{t})}{D^{t+1}(x^{t},y^{t})}\right]}^{\frac{1}{2}}$$

(8) Tfpch=Techch×Effch=Techch×Sech×Pech

*Tfpch* represents total factor productivity change index, *Techch* represents technological progress change index, *Effch* represents overall technical efficiency change index, Sech represents scale efficiency change index, Pech represents pure technical efficiency change index; ($x^{t}$, $y^{t}$) and ($x^{t+1}$, $y^{t+1}$) respectively represent the input-output vectors at period *t* and period *t* + 1; *M* > 1 indicates efficiency improvement; *M* = 1 indicates efficiency remains constant; *M* < 1 indicates efficiency decline (36).

### Factors affecting the efficiency of healthcare resource allocation in China

#### Research method and variable selection

The above analysis reveals that provinces and cities with relatively high efficiency include both economically developed and densely populated areas, as well as economically relatively underdeveloped and sparsely populated areas. Therefore, it is necessary to explore the factors that affect healthcare resource allocation efficiency (37). This study explores the factors influencing the efficiency of healthcare resource allocation in China from external and internal environments (38). The internal environment mainly considers the total amount of various healthcare resources and the fairness of healthcare resource allocation, while external environmental factors mainly consider the level of regional economic development, population distribution, and level of urbanization, as shown in Table 2. Based on the selection of influencing factors, nine indicators will be chosen as explanatory variables. The comprehensive efficiency of provinces and cities in different years will be taken as the dependent variable, and regression analysis will be conducted using the Tobit model, expressed as follows:

(11) $$\begin{array}{rl} Y_{it}= & \beta_{0}+\beta_{1}\ln gdp_{it}+\beta_{2}\ln pd_{it}+\beta_{3}\ln urban_{it}+\\ & \beta_{4}\ln people_{it}+\beta_{5}\ln bed_{it}+\beta_{6}\ln assets_{it}+\\ & \beta_{7}people_{it}+\beta_{8}bed_{it}+\beta_{9}assets_{it}+\varepsilon_{it} \end{array}$$

where, $Y_{it}$ represents comprehensive efficiency. Each variable subscripted by *i* corresponds to different decision-making units, namely the 31 provinces, autonomous regions, and municipalities directly under the central government of China, where *t* represents the observation year with values ranging from 2014–2019. $\beta_{0}$ represents the constant term, $\beta_{i}(i=1,2,\cdots ,9)$ stands for regression coefficient, and $\varepsilon_{it}$ denotes the residual term. The logarithm is taken for per capital GDP, population density, number of health technical personnel, number of beds, and total assets, aiming to mitigate the impact of dimensional differences.

**Table 2:** Selection of evaluation indicators for influencing factors.

Examining Factor	Specific Indicator	Symbol	Unit
Region Economic Development Level	Log of Per Capital GDP	lngdp	Yuan/person
Population Distribution	Log of Population Density	lnpd	Person/km^2^
Urbanization Level	Urbanization Rate	Urban	%
Total Human Health Resources	Log of Health Technical Personnel	lnpeople	%
Total Material Health Resources	Log of Beds	lnbed	Bed
Total Financial Health Resources	Log of Total Assets	lnassets	Yuan
Fairness of Human Resource Allocation	Theil Index of Health Technical Personnel	People	-
Fairness of Material Resource Allocation	Theil Index of Bed Numbers	Bed	-
Fairness of Financial Resource Allocation	Theil Index of Total Assets	Assets	-

## Results

### Analysis of fairness in healthcare resource allocation

This study calculated the Theil index of healthcare resource allocation in China from a population perspective using Stata 14 software, as shown in Table 3. From an overall balance perspective, the total Theil index of total assets is the highest, with values ranging from 0.0363–0.0478, indicating the poorest balance of healthcare resources by population distribution for total assets. The total Theil index of the number of beds is the lowest, with values ranging from 0.0074–0.0089, indicating relatively better balance.

**Table 3:** Theil index of China's healthcare resource allocation from population perspective, 2014–2019.

Indicator	Year	Overall Theil Index	Intra-regional Disparity		Inter-regional Disparity	
			Theil Index	Contribution Rate	Theil Index	Contribution Rate
Health Technical Personnel	2014	0.0108	0.0091	84.41%	0.0017	15.59%
	2015	0.0105	0.0090	85.52%	0.0015	14.48%
	2016	0.0105	0.0089	85.26%	0.0015	14.74%
	2017	0.0098	0.0081	82.18%	0.0018	17.82%
	2018	0.0095	0.0075	78.98%	0.0020	21.02%
	2019	0.0090	0.0073	80.37%	0.0018	19.63%
Practicing (assistant) physicians	2014	0.0113	0.0090	79.22%	0.0024	20.78%
	2015	0.0115	0.0090	78.66%	0.0024	21.34%
	2016	0.0115	0.0088	76.97%	0.0026	23.03%
	2017	0.0112	0.0085	75.35%	0.0028	24.65%
	2018	0.0119	0.0084	70.60%	0.0035	29.40%
	2019	0.0113	0.0079	69.74%	0.0034	30.26%
Registered Nurses	2014	0.0139	0.0119	85.23%	0.0021	14.77%
	2015	0.0135	0.0118	87.73%	0.0017	12.27%
	2016	0.0125	0.0110	87.72%	0.0015	12.28%
	2017	0.0111	0.0095	85.49%	0.0016	14.51%
	2018	0.0104	0.0086	82.80%	0.0018	17.20%
	2019	0.0100	0.0088	88.00%	0.0012	12.00%
Number of Beds	2014	0.0074	0.0063	85.99%	0.0010	14.01%
	2015	0.0075	0.0063	84.83%	0.0011	15.17%
	2016	0.0080	0.0068	84.69%	0.0012	15.31%
	2017	0.0083	0.0068	81.09%	0.0016	18.91%
	2018	0.0088	0.0069	78.03%	0.0019	21.97%
	2019	0.0089	0.0063	71.37%	0.0025	28.63%
Total Assets	2014	0.0478	0.0345	72.26%	0.0133	27.74%
	2015	0.0440	0.0336	76.27%	0.0104	23.73%
	2016	0.0414	0.0318	77.02%	0.0095	22.98%
	2017	0.0363	0.0286	78.68%	0.0077	21.32%
	2018	0.0374	0.0313	83.62%	0.0061	16.38%
	2019	0.0371	0.0319	86.08%	0.0052	13.92%

From the analysis of regional disparity contribution rates, the overall regional disparity contribution rates of healthcare resources for health technical personnel, practicing (assistant) physicians, registered nurses, total assets, and the number of beds is higher than the inter-regional disparity contribution rates, indicating that the differences in the allocation of these five types of healthcare resources mainly come from differences within the eastern, central, and western regions. The intra-regional disparity contribution rates of health technical personnel, practicing (assistant) physicians, registered nurses, and the number of beds show a decreasing trend overall, while the intra-regional disparity contribution rate of total assets shows an increasing trend year by year. The total assets have the highest intra-regional Theil index, indicating that the intra-regional allocation of total assets for healthcare resources is the most unfair.

### Static efficiency analysis based on DEA-BCC model

This study used DEAP 2.1 software to calculate the efficiency of healthcare resources allocation in China, as shown in Table 4. The results indicate that the efficiency of healthcare resources allocation in China from 2014–2019 has shown an overall increasing trend. The mean value in 2014 was 0.908, which increased to 0.932 in 2017, indicating a continuous improvement in the efficiency of healthcare resource allocation in China. Eight provinces and cities including Shanghai, Zhejiang, Jiangxi, Henan, and Guangxi maintained DEA effectiveness from 2014–2019, with Tibet considered effective, possibly due to insufficient resource input, indicating issues of inadequate investment in healthcare resources and structural imbalance in resource allocation (39). Hebei, Hunan, Guangdong, Chongqing, and Sichuan achieved DEA effectiveness for several consecutive years, indicating a reasonable utilization of healthcare resources. The number of provinces with DEA effectiveness overall shows an increasing trend, further demonstrating the full utilization of healthcare resources in various provinces and cities in China.

**Table 4:** Efficiency values of provinces and cities in China from 2014–2019.

Province/City	2014	2015	2016	2017	2018	2019
Beijing	0.925	0.95	0.995	0.95	0.988	1
Tianjin	1	1	0.996	0.973	0.994	1
Hebei	1	1	1	1	1	0.95
Shanxi	0.599	0.60	0.659	0.675	0.703	0.676
Inner Mongolia	0.645	0.64	0.67	0.69	0.711	0.665
Liaoning	0.703	0.71	0.731	0.728	0.73	0.726
Jilin	0.676	0.69	0.71	0.731	0.715	0.716
Heilongjiang	0.69	0.74	0.783	0.793	0.772	0.829
Shanghai	1	1	1	1	1	1
Jiangsu	0.91	0.93	0.923	0.939	0.904	0.896
Zhejiang	1	1	1	1	1	1
Anhui	0.908	0.91	0.915	0.936	0.94	0.931
Fujian	0.892	0.88	0.912	0.904	0.903	0.898
Jiangxi	1	1	1	1	1	1
Shandong	0.892	0.89	0.907	0.917	0.895	0.884
Henan	1	1	1	1	1	1
Hubei	0.962	0.95	0.94	0.964	0.978	1
Hunan	0.974	1	1	1	1	0.989
Guangdong	1	1	1	1	1	0.998
Guangxi	1	1	1	1	1	1
Hainan	0.923	0.93	0.95	0.961	0.957	0.938
Chongqing	0.996	1	1	1	0.972	1
Sichuan	0.984	1	1	1	0.995	1
Guizhou	1	1	1	1	1	1
Yunnan	1	1	1	1	1	1
Tibet	1	1	1	1	1	1
Shaanxi	0.809	0.84	0.843	0.861	0.883	0.839
Gansu	0.894	0.93	1	0.966	1	0.973
Qinghai	0.902	0.85	0.923	0.882	0.944	0.997
Ningxia	0.931	0.95	0.969	0.966	1	1
Xinjiang	0.932	0.98	0.992	0.971	0.957	1
Effective Number	11	13	14	13	13	15
Mean Value	0.908	0.915	0.93	0.929	0.934	0.932

Specifically, in 2019, China's overall efficiency in the allocation of healthcare resources was at a relatively high level, with an efficiency mean value of above 0.9, with pure technical efficiency and scale efficiency at 0.936 and 0.996, respectively. However, there were significant differences among provinces, autonomous regions, and municipalities directly under the central government, as shown in Table 5. In China, there are 15 provinces and cities that have achieved DEA-effective allocation of healthcare resources, where the input of resources leads to the maximum output, namely Beijing, Tianjin, Shanghai, Zhejiang, Jiangxi, Henan, Hubei, Guangxi, Chongqing, Sichuan, Guizhou, Yunnan, Tibet, Ningxia, and Xinjiang. Among the provinces and cities that are not DEA-effective, Shanxi and Inner Mongolia have efficiency values below 0.7, with Inner Mongolia having the lowest value at only 0.67. Additionally, it can be observed that the issues of low allocation efficiency and inadequate input scale coexist, meaning that China's allocation efficiency of healthcare resources is both technically and scale inefficient, with 11 provinces and cities in this state.

**Table 5:** Comprehensive efficiency, pure technical efficiency, and scale efficiency of healthcare resources in each province in 2019.

Province/City	Comprehensive Efficiency	Pure Technical Efficiency	Scale Efficiency	Returns to Scale
Beijing	1	1	1	-
Tianjin	1	1	1	-
Hebei	0.95	0.95	0.999	drs
Shanxi	0.676	0.676	1	-
Inner Mongolia	0.665	0.666	0.999	irs
Liaoning	0.726	0.731	0.994	irs
Jilin	0.716	0.719	0.997	drs
Heilongjiang	0.829	0.833	0.995	irs
Shanghai	1	1	1	-
Jiangsu	0.896	0.909	0.985	drs
Zhejiang	1	1	1	-
Anhui	0.931	0.931	0.999	irs
Fujian	0.898	0.899	1	-
Jiangxi	1	1	1	-
Shandong	0.884	0.946	0.934	drs
Henan	1	1	1	-
Hubei	1	1	1	-
Hunan	0.989	1	0.989	drs
Guangdong	0.998	1	0.998	drs
Guangxi	1	1	1	-
Hainan	0.938	0.939	0.999	irs
Chongqing	1	1	1	-
Sichuan	1	1	1	-
Guizhou	1	1	1	-
Yunnan	1	1	1	-
Tibet	1	1	1	-
Shaanxi	0.839	0.841	0.997	irs
Gansu	0.973	0.975	0.998	drs
Qinghai	0.997	1	0.997	drs
Ningxia	1	1	1	-
Xinjiang	1	1	1	-
Mean Value	0.932	0.936	0.996	

irs represents increasing returns to scale, drs represents decreasing returns to scale,—indicates constant returns to scale.

Pure technical efficiency refers to achieving the relative optimal output with the existing inputs under variable returns to scale conditions (40). In 2019, there were a total of 18 provinces and cities in China that were pure technically efficient, meaning that these provinces and cities effectively utilized the resources invested and achieved relatively optimal outputs. Scale efficiency refers to the ability to achieve maximum output while using different factor proportions under existing supply conditions. For provinces that are not scale efficient, the results show that 8 provinces and cities exhibited a decreasing trend, indicating that the current allocation of healthcare resources in these provinces exceeds a reasonable level and requires appropriate control of inputs. Other provinces, on the other hand, were able to fully utilize healthcare resources under the existing scale.

### Analysis of non-DEA effective projection values

To further analyze the issue of input redundancy or insufficient output in non-DEA efficient decision-making units, this study conducted a projection analysis of input-output quantities based on the efficiency measurement results of each province in 2019, as shown in Tables 6, 7. According to Table 6, the investment in healthcare resources in Hebei, Liaoning, Jiangsu, Fujian, Shandong, and Hainan in the eastern region shows an excess in human, material, and financial resources. The four provinces in the central region all show varying degrees of overallocation in the allocation of the five input resources, with the most significant redundancy being in the allocation of health technical personnel. The three provinces in the western region also demonstrate varying degrees of redundancy in the allocation of the five input resources, with Inner Mongolia showing the most severe redundancy across all input indicators, followed by Shaanxi. For detailed information on the redundancy rates and values, please refer to Table 6.

**Table 6:** Input redundancy in non-pure technically efficient provinces.

Region	Decision-making Unit	Project	Input Index				
			X1	X2	X3	X4	X5
Eastern Region	Hebei	Redundancy Value	75,689.98	68,300.92	9,208.702	50,744.26	476.746
		Redundancy Rate	15.44%	29.88%	4.98%	11.80%	16.22%
	Liaoning	Redundancy Value	83,244.67	41,872.3	39,982.79	100,072.4	487.496
		Redundancy Rate	26.92%	33.80%	28.74%	31.89%	26.92%
	Jiangsu	Redundancy Value	57,420.61	37,003.05	25,371.66	46,785.1	631.839
		Redundancy Rate	9.07%	14.53%	9.07%	9.07%	14.17%
	Fujian	Redundancy Value	26,680.62	11,927.44	13,529.49	20,496.6	172.228
		Redundancy Rate	10.14%	11.99%	11.64%	10.14%	10.14%
	Shangdong	Redundancy Value	121,144.1	62,902.19	56,278.84	47,452.86	231.948
		Redundancy Rate	15.49%	19.95%	16.49%	7.54%	5.41%
	Hainan	Redundancy Value	8,428.908	1,460.809	6,628.854	5,824.02	27.742
		Redundancy Rate	12.45%	6.11%	20.68%	11.70%	6.11%
Central Region	Shanxi	Redundancy Value	88,977.22	47,627.29	35,285.32	79,708.7	418.362
		Redundancy Rate	34.51%	45.04%	32.37%	36.49%	32.37%
	Jilin	Redundancy Value	55,716.36	31,095.09	22,326.94	47,947.95	342.321
		Redundancy Rate	29.56%	39.35%	28.15%	28.15%	29.20%
	Heilongjiang	Redundancy Value	49,811.2	25,166.26	16,310.85	75,569.45	252.36
		Redundancy Rate	20.96%	26.91%	16.71%	28.78%	16.71%
	Anhui	Redundancy Value	24,749.11	14,021.23	14,598.12	29,470.09	153.48
		Redundancy Rate	6.85%	10.13%	8.93%	8.48%	6.85%
Western Region	Inner Mongolia	Redundancy Value	71,313.08	33,129.39	26,875.28	53,822.4	438.102
		Redundancy Rate	36.31%	42.42%	33.41%	33.41%	37.46%
	Shanxi	Redundancy Value	87,002.69	17,293.98	31,804.16	42,296.38	419.988
		Redundancy Rate	24.59%	15.91%	21.14%	15.91%	23.02%
	Gansu	Redundancy Value	6,095.491	3,609.586	2,008.202	26,714.37	23.599
		Redundancy Rate	3.41%	5.75%	2.53%	14.75%	2.53%

**Table 7:** Output deficiency situation of provinces with non-pure technical efficiency.

Region	Decision-making Unit	Project	Output Index		
			Y1	Y2	Y3
Eastern Region	Hehei	Insufficient Value	0	0	0
		Insufficient Rate	0.00%	0.00%	0.00%
	Liaoning	Insufficient Value	5.356	0	0
		Insufficient Rate	6.77%	0.00%	0.00%
	Jiangsu	Insufficient Value	2.457	0	0
		Insufficient Rate	2.79%	0.00%	0.00%
	Fujian	Insufficient Value	0	0	0
		Insufficient Rate	0.00%	0.00%	0.00%
	Shangdong	Insufficient Value	6.344	0	0
		Insufficient Rate	7.29%	0.00%	0.00%
	Hainan	Insufficient Value	0	0	0
		Insufficient Rate	0.00%	0.00%	0.00%
Central Region	Shanxi	Insufficient Value	0	24,55,444.44	0
		Insufficient Rate	0.00%	1.97%	0.00%
	Jilin	Insufficient Value	0	0	0
		Insufficient Rate	0.00%	0.00%	0.00%
	Heilongjiang	Insufficient Value	7.633	36,09,0354.44	0
		Insufficient Rate	9.29%	25.56%	0.00%
	Anhui	Insufficient Value	2.536	0	0
		Insufficient Rate	2.96%	0.00%	0.00%
Western Region	Inner Mongolia	Insufficient Value	1.419	0	0
		Insufficient Rate	1.95%	0.00%	0.00%
	Shanxi	Insufficient Value	2.877	0	0
		Insufficient Rate	3.40%	0.00%	0.00%
	Gansu	Insufficient Value	0	0	0
		Insufficient Rate	0.00%	0.00%	0.00%

Output deficiency refers to the part where the target output indicator value is higher than the actual value, indicating that the existing output has not been able to meet the demand. According to Table 7, the three provinces in the eastern region have varying degrees of output deficiency in the “bed utilization rate,” with Shandong having the largest output deficiency at 6.344%, requiring a further increase of 7.29% to reach the target value. The three provinces in the central region also have varying degrees of output deficiency in the “bed utilization rate,” with Heilongjiang experiencing output deficiency in the “number of outpatient visits.” The two provinces in the western region have varying degrees of output deficiency in the “bed utilization rate,” with Shaanxi having the largest output deficiency in the “bed utilization rate.”

### Dynamic efficiency analysis based on DEA-Malmquist model

This paper utilizes the Malmquist model to analyze the dynamic changes in total factor productivity of China's healthcare resources and their decomposition, obtaining the results of total factor efficiency changes in different years and regions from 2014–2019, as shown in Tables 8, 9. From a longitudinal perspective, the mean value of the dynamic changes in total factor productivity is 0.965 < 1, indicating an overall downward trend in total factor productivity. Looking at the annual changes, total factor productivity showed an upward trend from 2014–2016, followed by a decline. The changes in decomposition index show differences in the trends of various factors between years, with technical efficiency showing an initial increase followed by a decrease and then another increase followed by a decrease; technological progress initially increases, then decreases after 2017 before increasing again; and scale efficiency shows an initial increase followed by a decrease and then an increase. The mean value of the technological progress index is less than 1, indicating that changes in technology play a major role in the changes in total factor productivity. Therefore, improving technological levels can achieve steady growth in the operational efficiency of the healthcare system.

**Table 8:** Total factor productivity indicators and decomposition of China's healthcare resources from 2014–2019.

Year	Effch	Techch	Pech	Sech	Tfpch
2014–2015	1.008	0.935	1.007	1.001	0.942
2015–2016	1.018	0.963	1.012	1.006	0.981
2016–2017	1.001	0.973	1.002	0.998	0.974
2017–2018	1.005	0.957	1.003	1.002	0.961
2018–2019	0.998	0.97	0.993	1.005	0.968
Mean Value	1.006	0.96	1.004	1.002	0.965

**Table 9:** Total factor productivity indicators and their decomposition of healthcare resources in various regions of China from 2014–2019.

Region	Province	Effch	Techch	Sech	Pech	Tfpch	Rank
Eastern Region	Beijing	1.016	0.986	1.014	1.002	1.002	1
	Tianjin	1	0.968	1	1	0.968	15
	Hebei	0.99	0.956	0.99	1	0.946	27
	Liaoning	1.006	0.969	1.008	0.999	0.975	11
	Shanghai	1	0.977	1	1	0.977	8
	Jiangsu	0.997	0.979	0.996	1.001	0.976	9
	Zhejiang	1	0.974	1	1	0.974	13
	Fujian	1.002	0.973	1.002	1	0.975	11
	Shangdong	0.998	0.958	0.989	1.009	0.956	23
	Guangdong	1	0.968	1	1	0.967	16
	Hainan	1.003	0.94	0.992	1.011	0.943	28
Central Region	Shanxi	1.025	0.938	1.025	1	0.961	22
	Jilin	1.012	0.973	1.012	0.999	0.984	4
	Heilongjiang	1.037	0.958	1.038	0.999	0.993	3
	Anhui	1.005	0.977	1.003	1.002	0.981	6
	Jiangxi	1	0.962	1	1	0.962	21
	Henan	1	0.939	1	1	0.939	29
	Hubei	1.008	0.964	1	1.008	0.971	14
	Hunan	1.003	0.949	1	1.003	0.952	25
Western Region	Guangxi	1	0.947	1	1	0.947	26
	Inner Mongolia	1.006	0.97	1.006	1	0.976	9
	Chongqing	1.001	0.977	1	1.001	0.978	7
	Sichuan	1.003	0.964	1	1.003	0.967	16
	Guizhou	1	0.937	1	1	0.937	30
	Yunnan	1	0.964	1	1	0.964	20
	Tibet	1	0.888	1	1	0.888	31
	Shanxi	1.007	0.988	1.008	0.999	0.995	2
	Gansu	1.017	0.951	1.012	1.005	0.967	16
	Qinghai	1.02	0.938	1.005	1.015	0.956	23
	Ningxia	1.014	0.953	1	1.014	0.967	16
	Xinjiang	1.014	0.969	1.011	1.003	0.983	5
Mean Value		1.006	0.96	1.004	1.002	0.965	

According to the data in Table 9, only Beijing in China has a total factor productivity index greater than 1, while the total factor productivity indices of other provinces are less than 1. This indicates that the level of total factor productivity is in a state of regression, meaning that the efficiency of healthcare resource allocation in China is declining. A possible reason for this is that the technological progress index values of healthcare resources in various provinces and cities in China are all less than 1, suggesting that the relatively low technological progress index has placed a certain constraint on the improvement of healthcare resource allocation efficiency (41).

### Empirical results analysis of factors affecting the efficiency of healthcare resource allocation

This study used Stata 14 software to estimate the Tobit model, and the empirical results are shown in Table 10. The results indicate that population density, urbanization rate, number of beds, total assets, fairness of health technical personnel allocation, and fairness of total asset allocation significantly affect the efficiency of healthcare resource allocation in various provinces of China. Specifically, population density, total assets, and fairness of total asset allocation are positively correlated with the comprehensive efficiency of healthcare resources in each province, while urbanization rate, number of beds, and fairness of health technical personnel allocation are negatively correlated with the comprehensive efficiency of healthcare resources in each province.

**Table 10:** Tobit regression results of factors influencing the efficiency of healthcare resource allocation in China.

Explanation Variable	Regression Coefficient	Standard Error	*t* Value	*P* Value
lngdp	0.054	0.040	1.34	0.18
lnpd	0.046	0.008	5.96	<0.001***
urban	−1.028	0.126	−8.14	<0.001***
lnpeoper	−0.051	0.094	−0.55	0.585
lnbed	−0.177	0.075	−2.34	0.02**
lnassets	0.227	0.051	4.46	<0.01***
people	−0.003	0.001	−2.87	0.005***
bed	0.000	0.001	0.53	0.6
assets	0.001	0.001	2.41	0.017**

**, *** represent significance levels of 5%, and 1% respectively.

## Discussion

### The impact of regional development level on the efficiency of healthcare resource allocation

From Table 10, it can be observed that at a significance level of 1%, population density has a positive impact on the efficiency of healthcare resource allocation in China, indicating that regions with higher population density tend to have higher efficiency in healthcare resource allocation. Urbanization rate at a significance level of 1%, on the other hand, has a negative impact on the efficiency of healthcare resource allocation in various provinces of China, meaning that an increase in urbanization rate leads to a decrease in the efficiency of healthcare resource allocation. This result is consistent with the findings of Xu Xiaofang, et al. (42). The reason for this phenomenon may be that as some of the population gradually moves into cities, it drives an increase in the supply of resources in urban areas, while the development of healthcare services in towns and villages lags behind, exacerbating the disparity in the level of healthcare development between urban and rural areas, leading to a decrease in resource allocation efficiency in China.

### The impact of the total amount of healthcare resources on the efficiency of healthcare resource allocation

From Table 10, it can be seen that the number of health technical personnel does not play a significant role in the efficiency of healthcare resource allocation in China. This may be because an excessive number of health technical personnel leads to resource redundancy and surplus, resulting in resources not being utilized for production at the minimum cost, thus having a negative impact. However, if the allocation of health technical personnel is fair, the efficiency of allocation will also improve, leading to a neutralizing effect, thereby the significant role in the efficiency of healthcare resource allocation in China not being very obvious. The number of beds at the 5% significance level has a negative impact on the efficiency of healthcare resource allocation in China, possibly due to an excessive number of beds resulting in bed vacancies and underutilization potential, which leads to low efficiency in resource allocation (43). This is consistent with the reality of developed regions having relatively surplus healthcare resources while underdeveloped regions lack sufficient healthcare resource allocation. At the 1% significance level, total assets have a positive impact on the efficiency of healthcare resource allocation in China, meaning the higher the investment in total assets, the higher the efficiency of resource allocation, possibly because increasing investment in total assets can provide sufficient funding for medical institutions to improve their technical levels, promote the sustainable development of medical institutions, and enhance the efficiency of healthcare resource allocation, which is consistent with the results of Yan et al. (44).

### The impact of fairness in the allocation of healthcare resources on the efficiency of healthcare resource allocation

From Table 10, it can be seen that at a 1% significance level, the Theil index of health technical personnel has a negative impact on the efficiency of healthcare resource allocation in China. That is, the smaller the Theil index of health technical personnel, the fairer the allocation of health technical personnel, and the higher the efficiency of healthcare resource allocation. It can be observed that the fairness in the allocation of health technical personnel has the greatest and positive impact on the efficiency of healthcare resource allocation, therefore requiring special attention. The Theil index of the number of beds does not have a significant impact on the efficiency of healthcare resource allocation in China, indicating that fair allocation of healthcare bed resources does not necessarily improve the efficiency of healthcare service utilization. The Theil index of total assets at a 5% significance level has a positive impact on the efficiency of healthcare resource allocation in China, meaning that the larger the Theil index of total assets, the more unfair the allocation of healthcare resources, and the higher the efficiency of healthcare resource allocation. Asset inequality might boost efficiency in some cases. For example, resource concentration can lead to economies of scale by directing healthcare resources to specific areas or institutions. Also, asset inequality might reflect varying regional demands for healthcare. Regions with higher demand may need more resources, and this demand—matching effect can enhance resource use efficiency (45). This suggests that the fairness of allocation of total healthcare assets may to a certain extent reduce its utilization efficiency, which is an important finding of this study. It may be due to the convergence of the imbalance in healthcare resource allocation and the uneven demand caused by large-scale population movements.

## Conclusion

In order to investigate the impact of fairness in healthcare resource allocation on its utilization efficiency, this study first used the Theil index to measure the fairness of healthcare resource allocation, then used the DEA data envelopment analysis to assess the efficiency of healthcare resource allocation. Then the Tobit model was used to analyze the impact of fairness in healthcare resource allocation and other influencing factors on the efficiency of healthcare resource allocation. This study provides a certain complementary role and theoretical significance to related fields, focusing on demonstrating that fairness in healthcare resource allocation does indeed influence the utilization efficiency of healthcare services to a certain extent. Specific conclusions include: (1) Disparities in the fairness of healthcare resource allocation in China are mainly reflected within regions, especially evident in the regional disparities of total assets. (2) Overall, the efficiency of healthcare resource allocation in China is improving. In 2019, there were 15 provinces and cities in China with effective DEA healthcare resource allocation, while 11 provinces had simultaneous inefficiencies in both technical and scale aspects. Low technological level is a key factor affecting the efficiency of healthcare resource allocation in China. (3) Through the study of factors influencing the efficiency of healthcare service utilization, it was found that population density, urbanization rate, number of hospital beds, total assets, fairness in the allocation of health technical personnel, and fairness in the allocation of total assets have significant impacts on the efficiency of healthcare resource allocation in various provinces in China. Specifically, population density, total assets, and fairness in the allocation of total assets are positively correlated with the overall efficiency of healthcare resource allocation in each province, while urbanization rate, number of hospital beds, and fairness in the allocation of healthcare technical personnel are negatively correlated. The most important finding is that the fairness in the allocation of health technical personnel has the greatest and positive impact on the efficiency of healthcare resource allocation, and it is pointed out innovatively that fairness in the allocation of total healthcare assets may to a certain extent reduce their utilization efficiency.

In addition to having certain theoretical significance, the conclusions drawn in this article can also provide a certain reference and guidance for the formulation and practice of healthcare resource allocation policies to a certain extent. Based on this, the following suggestions are proposed in this article:

First, leverage the radiation effect of high-quality resources to optimize resource allocation within the region. The total amount of healthcare resources continues to increase, with significant differences within the region. The contribution rate of healthcare resource allocation within regions in China is greater than that between regions, indicating that internal regional differences are the main factor leading to the unfair allocation of healthcare resources in China. Among them, the regional differences in total assets are greater than those of other types of healthcare resources, further indicating significant differences among provinces and cities in the comprehensive strength of healthcare institutions in equipment, beds, construction area and so on. This phenomenon may be attributed to the focus on economically developed provinces and cities within regions during economic development, while neglecting other surrounding areas, and even sacrificing the interests of those in marginal areas (46), which is consistent with the research conclusions of Wang Xiaoyu and others (47). Therefore, it is suggested to establish a pyramid-shaped medical service system within the region, accelerate the construction of a complete medical service network, improve the regional coverage of healthcare resources (48), leverage the radiation effect of high-quality resources, harness the clustering effect of resource allocation, focus on internal regional synergies, and improve the quality and efficiency of resource allocation within the region.

Second, enhance regional healthcare planning efforts to reduce unnecessary resource allocation. The efficiency of healthcare resource allocation in China is on the rise, with significant differences in resource allocation efficiency among provinces and cities. Regional healthcare planning refers to a comprehensive plan for healthcare development and resource development within the region, which rationalizes the allocation of healthcare resources based on the health status and medical needs of the population within the region, and strategically arranges healthcare institutions of different levels, functions, and scales to balance the supply and demand of healthcare resources, thereby ensuring the full utilization of healthcare resources (49). Among the provinces and cities that were not effective in DEA in 2019, 8 provinces and cities were in the stage of decreasing returns to scale, indicating that their large scale would lead to lower scale efficiency, thus affecting the overall efficiency of healthcare resource allocation. For provinces and cities experiencing decreasing returns to scale, appropriate control of their input scale should be implemented. The projection analysis of non-DEA-effective provinces and cities shows that there is an excess phenomenon in the number of health technical personnel, practicing (assistant) physicians, registered nurses, beds, and total health expenses, indicating an excessive allocation of healthcare resources in non-DEA-effective provinces and cities. Therefore, provinces and cities should tailor measures according to local conditions, and reduce unnecessary resource allocation based on the results of input redundancy to achieve higher resource allocation efficiency at the minimum cost.

Thirdly, strengthen the construction of the healthcare talent team and improve the technological level of medical services. Total factor productivity is influenced by the index of technological progress, indicating that changes in technological level play a major role in the changes of total factor productivity. Research has found that increasing tangible input to leverage scale effects can only improve comprehensive technical efficiency, with limited impact on total factor productivity. A lower technological level is a major factor affecting the efficiency of healthcare resource allocation in China. The pure technical efficiency values and scale efficiency values of healthcare resource allocation in each province and city from 2014–2019 are relatively high, but due to the relatively low level of technological progress, the improvement in the operational efficiency of provincial and city medical service systems is not optimistic, which is consistent with the research conclusions of Shao and others (50). Therefore, medical institutions should increase investment to promote medical technological progress, strengthen the construction of the healthcare talent team, rely on talent for research and development innovation, accelerate the transformation of technological achievements, promote suitable healthcare technologies, enhance the technological level of medical service, and improve the efficiency of healthcare resource allocation in China.

Of course, this article also has certain limitations and deficiencies, which are hereby put forward for further discussion among peers: First, this article mainly discusses the fairness and efficiency of healthcare resource allocation at a macro level using panel data, lacking evidence support at the micro level; Second, this article only demonstrates that the fairness of healthcare resource allocation has a certain impact on utilization efficiency, lacking more robust causal relationship arguments; Third, due to the unavailability of relevant data, this article did not incorporate more comprehensive variable types including regional population health variables, thus failing to form a complete logical loop of economic development—resource quantity—fairness—efficiency—health. The above limitations and deficiencies will also be the direction of further research in the future.

## Data Availability

The original contributions presented in the study are included in the article/Supplementary Material, further inquiries can be directed to the corresponding author.
